# Risk factors of pleural effusion in post-liver transplant pediatric population

**DOI:** 10.12669/pjms.42.4.14742

**Published:** 2026-04

**Authors:** Muhammad Saqib, Nazia Iqbal, Ahmad Saqib, Safia Khan

**Affiliations:** 1 Muhammad Saqib, FCPS., PKLI & RC, Lahore, Pakistan; 2 Nazia Iqbal, FCPS., PKLI & RC, Lahore, Pakistan; 3 Ahmad Saqib, 2^nd^ year MBBS Student. Shalamar Medical & Dental College, Lahore, Pakistan; 4 Safia Khan, FCPS., PKLI & RC, Lahore, Pakistan

**Keywords:** Complications, Liver, Pleural effusion, Pediatric, Post-transplant, Pulmonary complications

## Abstract

**Background and Objective:**

Pediatric liver transplantation (LT) is curative therapy for end-stage liver disease. One of the most frequent pulmonary complications after LT in children is pleural effusion. With improvement in surgical techniques and postoperative intensive care, long-term survival is now more than 80%. This study was performed to assess the incidence, risk factors, and clinical significance of post-LT pleural effusion in children.

**Methodology:**

This retrospective cohort study from a single center enrolled 60 children who had undergone liver transplantation in our center from July 2021 to April 2024. Preoperative, perioperative, intraoperative, and postoperative parameters were collected and measured to determine factors related to the development of pleural effusion. Data was entered and analyzed in SPSS ver: 26.0.

**Results:**

Post-liver transplant (LT) pleural effusion occurred in 36/60 pediatric patients (60%). Some important risk factors were found in those patients who had pleural effusion. These were a Pediatric End-Stage Liver Disease (PELD) score of ≥18, malnutrition, and lower preoperative fibrinogen, albumin, and hemoglobin levels. A binary logistic model was used to evaluate risk factors for post operative pleural effusion among patients with postoperative fibrinogen, albumin, protein, INR, APTT, platelets, and Hemoglobin level. Odds ratio for Serum albumin level (B) was 34.545 for developing plural effusion.

**Conclusion:**

Pleural effusion is a frequent postoperative complication in children undergoing liver transplantation and is correlated with greater morbidity, especially in those patients necessitating therapeutic drainage. Preoperative and intraoperative factors can also act as significant predictors and prognostic markers. Early recognition and treatment of these risk factors may be helpful in outcomes.

## INTRODUCTION

Liver transplant (LT) is the ultimate management of end-stage liver disease (ESLD) and acute liver failure. Nevertheless, pulmonary complications are still a leading cause of morbidity and mortality after LT, particularly in children.^1^ The complications are correlated with increased mechanical ventilation duration, longer intensive care unit (ICU) stay, hospitalization time, and considerable decrease in short-term survival rates.^2^

Several risk factors have been shown to predispose children to post-transplant pulmonary complications.^3^ They are age, prior history of pulmonary disease, intraoperative acute respiratory failure necessitating mechanical ventilation, high Model for End-Stage Liver Disease (MELD) or Pediatric End-Stage Liver Disease (PELD) score, positive intraoperative fluid balance, extensive blood product transfusions, hypoalbuminemia, and the etiology and severity of the underlying liver disease.^4^,^5^

The Model for End-Stage Liver Disease (MELD) score is based on serum bilirubin, creatinine, and the international normalized ratio (INR) providing a continuous variable that is a very accurate predictor of 90 days mortality in patients with chronic liver diseases in children age 12 years or above.^6^ The Pediatric End-stage Liver Disease (PELD) score is intended to determine priority for children awaiting liver transplant under the age of 12 years.The five parameters used in the pediatric end-stage liver disease (PELD) score, are serum albumin, patient’s age at listing, international normalization ratio (INR), total bilirubin, and growth failure.^7^

Among these, pleural effusion is seen most frequently in the first week after transplant and usually on the right side. Most are mild and self-limited, but a proportion of them may necessitate therapeutic measures like thoracentesis or chest tube placement.^8^ Large-volume effusions or recurrences can cause atelectasis and impaired respiratory function, especially in infants, thus complicating the postoperative course.^9^

This research was done to establish the incidence of post-LT pleural effusion, determine risk factors associated with it, evaluate the requirement for therapeutic intervention, and assess its influence on early postoperative results in a children’s liver transplant unit.

Over the past few years, the perioperative management of fluid has evolved from liberal to restrictive strategies. The evolution is based on normal parameters such as blood pressure, central venous pressure, heart rate, and urine output, and can potentially play an important role in the prevention of complications, including pleural effusion.

### Study rationale:

Despite substantial literature from developed countries, critical knowledge gaps exist specially if we talk about the respiratory complication like pleural effusion in post pediatric liver transplant in South Asian region. From Pakistan there is no large published data found on this topic. Given the distinct epidemiological, clinical and resource context of lower middle income countries (LMIC) transplant programs, extrapolation from high-income country data may not adequately reflect the local clinical decision making.

This study addresses these gaps by providing the systematically collected data on post liver transplant pleural effusion in pediatric population in Pakistan. Our results help in developing evidence-based prevention of risk factors of pleural effusion in post LDLT pediatric recipient in LMIC.

## METHODOLOGY

This retrospective cohort study was conducted in the Pediatric Intensive Care, PKLI & RC, Lahore Pakistan.

### Ethical Approval:

It was obtained from the Institutional Review Board Ref No. PKLI-IRB/AP/00362024; dated July 4, 2024. The waiver of consent was done due to the retrospective nature of the study and since no direct intervention was involved. All personal information about the patients was kept strictly confidential.

We retrospectively analyzed the data for pediatric patients ranging from six months to 15 years who underwent living donor liver transplant at PKLI & RC from July 2021 to April 2024. For the period of time described, a total of 60 pediatric recipients of liver transplant were included in the study. Data collection was performed by means of a pre-designed proforma with the main emphasis on post-liver transplant pleural effusion.

At our center, plain chest X-rays were performed daily until the patients were successfully extubated and remained free of pulmonary symptoms. Advanced imaging modalities like CT/MRI and lung ultrasonography were used only when clinically indicated.

Demographic data collected included baseline patient characteristics, Complications related to end-stage liver disease, preoperative pulmonary comorbidities, serum albumin levels, and other relevant laboratory parameters were recorded. Intraoperative blood loss, volume of fluid infusion, urine output, and volume of blood product transfusion, perioperative fluid retention, and fluid balance were the perioperative variables recorded.

Postoperative data included the incidence and management for pleural effusion, including any therapeutic intervention required. If appropriate, the characteristics of the pleural fluid and duration of drainage were noted. Postoperatively, all patients were admitted to the PICU and remained intubated until such a time that extubation was clinically judged appropriate, after which time they were transferred to the pediatric ward following hemodynamic stabilization.

### Statistical Analysis:

Data analysis was done using SPSS, version 26. Descriptive statistics were used to summarize the dataset. Categorical variables include malnutrition, pre-existing lung disease, presence of pleural effusion, need for therapeutic intervention, and gender, which were reported as frequencies and percentages. Continuous variables, including age and PELD/MELD score, duration of PICU stay, and intraoperative fluid retention was presented as mean ± SD or median with IQR depending on the data distribution. Normality for quantitative variables was tested using the Shapiro-Wilk statistical test. Paired t test was used to assess mean difference among pre and post biochemistry. Binary logistic regression model was used to assess risk factors for pleural effusion and post operative biochemistry of patients. A p < 0.05 was used to assess statistical significance.

## RESULTS

Records of sixty pediatric patients who underwent liver transplantation between July 2021 and April 2024 were reviewed. Patient demographics and clinical/laboratory characteristics are summarized in Table-I.

**Table-I:** Demographic, clinical, and laboratory parameters of pediatric liver transplant recipients (n = 60).

Variables n=60	Categories	Frequency	Percentage
Gender	Male	40	66.7
	Female	20	33.3
Age (years) Mean =5.55 ± 3.97	< 5 years	27	45.0
	5-10 years	24	40.0
	> 10 years	9	15.0
Weight Percentile	< 5th Centile	28	46.7
	≥ 5th Centile	32	53.3
Pre-existing Lung Disease	Yes	6	10.0
	No	54	90.0
PELD/MELD Score	< 25	48	80.0
	25-29	4	6.7
	30-34	5	8.3
	≥ 35	3	5.0
Pre-operative Fibrinogen (mg/dL)	< 100	4	6.7
	100-200	25	41.7
	201-400	29	48.3
	> 400	2	3.3
Post-operative Fibrinogen (mg/dL)	< 100	11	18.3
	100-200	15	25.0
	201-400	7	11.7
	> 400	0	0.0
	Not Assessed	27	45.0
Pre-operative Albumin (g/dL)	< 2.5	9	15.0
	≥ 2.5	51	85.0
Post-operative Albumin (g/dL)	< 2.5	26	43.3
	≥ 2.5	34	56.7
Intraoperative Fluid Balance	< 100 mL/kg	30	50.0
	> 100 mL/kg	30	50.0
Hemoglobin Levels	Pre-operative < 10 g/dL	22	36.7
	Pre-operative ≥ 10 g/dL	38	63.3
	Post-operative < 10 g/dL	40	66.7
	Post-operative ≥ 10 g/dL	20	33.3
Outcome	Discharged	50	83.3
	Death	10	16.7

Cirrhosis secondary to biliary atresia was the most common indication for liver transplantation, observed in 17 patients (28.3%), followed by progressive familial intrahepatic cholestasis in 15 patients (25%). A total of 59 patients (98.3%) received partial liver grafts; predominantly the left lateral segment of the left lobe; from living donors, while one patient received a whole liver graft from a cadaveric donor.

The majority of patients were male (40/60; 66.6%). Twenty seven patients (45%) were under five years of age, including five (18.5%) who were less than one year old. The mean age of the cohort was 5.5 years, ranging from seven months to 14 years.

Post-transplant pleural effusion was observed in 36 patients (60%), predominantly on the right side (35/36; 97.2%). Fourteen patients (23.3%) required therapeutic interventions such as thoracentesis or chest tube placement. Tracheostomy was done in three patients (5%), and 15 patients (25%) required reintubation due to complications including convulsions, lung collapse, Surgical complications, including sepsis.

Patients developing pleural effusion typically had lower total protein levels and serum albumin levels. Preoperative serum albumin levels below 2.5 g/dL were documented in nine patients (15%), while postoperative levels were below this threshold in 26 patients (43.3%). Most of the patients, 52/60 (86.6%), had a PELD/MELD score of <25; eight patients(13.3%) had scores ≥30. Malnutrition, as defined by operational criteria, was present in 28 patients (46.6%). Only six patients (10%) had pre-existing pulmonary disease.

In the preoperative workup, fibrinogen levels ranged from 150 to 400 mg/dL in 44 patients (73.3%). Postoperatively, 11 patients (18.3%) had fibrinogen levels <100 mg/dL, and these patients more often developed significant pleural effusion. Fibrinogen was not measured postoperatively in five patients (8.3%), and 27 patients (45%) were not assessed for postoperative fibrinogen due to the absence of clinical indications includes clinically overt bleeding, deranged coagulation, and DIC. Of those tested postoperatively, most had fibrinogen levels between 100-200 mg/dL.

Preoperative hemoglobin was <10 g/dL in 22 patients (36.6%), while 40 patients (66.6%) had a post-transplant hemoglobin less than 10 g/dL. This contributed to frequent transfusion requirements and was associated with a higher incidence of Pleural effusion.

Intraoperative fluid balance >100 mL/kg was observed in 30 patients (50%) and associated with longer PICU lengths of stay, higher oxygen needs and a higher incidence of acute kidney injury independent of age or baseline illness severity.

The mean duration of PICU stay and mechanical ventilation was 11.13 ± 12.84. days and 5.81 ± 9.3 days, respectively. Overall post-transplant survival was 83.3%, which includes 50 successfully discharged patients. Causes of death included primary graft non-function (n = 1), bacterial pneumonia (n = 1), and septicemia (n = 8). No mortality was directly attributable to pleural effusion. (Table-I).

Paired t test was used to assess pre and post biochemistry of patients. Mean difference was significant among fibrinogen, albumin, total protein, platelets, Hemoglobin and BUN Levels. (Table-II). A binary logistic model was used to evaluate risk factors for post operative pleural effusion among patients with postoperative fibrinogen, albumin, protein, INR, APTT, platelets, and Hemoglobin level . Odds ratio for Serum albumin level (B) was 34.545 for developing pleural effusion. (Table-III).

**Table-II:** Pre and Post Operation Biochemistry of patients.

Biochemistry	Mean	Std. Deviation	Sig. (2-tailed)
Pre-OP Fibrinogen - Post-OP Fibrinogen	109.048	87.616	000
Pre-OP albumin - Post-OP Albumin	.59500	.75247	.000
Pre-OP Total Protein - Post-OP Total Protein	2.48813	.83466	.000
Pre-OP Platelets - Post-OP Platelets	80.53125	107.02004	.000
Pre-OP Hb - Post-OP HB	1.56563	1.91673	.000
Pre-OP INR - Post-OP INR	-.32156	.72348	.017
Pre-OP APTT - Post-OP APTT	-1.03750	17.45732	.739
Pre-OP BUN - Post-OP BUN	-8.12125	16.25447	.008
Pre-OP Creatinine - Post-OP Creatinine	-.06750	.98558	.701

**Table-III:** Binary logistic analysis of risk factor for pleural infusion

Classification Table^a^					
Observed			Predicted		
			Post-Op Plueral Effusion		Percentage Correct
			Yes	No	
Step 1	Post-Op Plueral Effusion	Yes	20	0	100.0
		No	0	1	100.0
	Overall Percentage				100.0

a. The cut value is .500

## DISCUSSION

Despite conservative management of underlying diseases, optimization of organ allocation principles, improvements in transplantation techniques, increased practice of living donation, and perioperative and postoperative care, pediatric organ transplantation remains a complex challenge, requiring a high level of multidisciplinary practice and experience.^1^ Children may experience respiratory complications as their liver disease worsens. Portal hypertension may be linked to hepatopulmonary syndrome, which lowers patient quality of life and increases mortality. Portopulmonary hypertension is another effect that could be linked to a lower chance of survival. Clinicians must be able to identify when patients should be sent to a pediatric pulmonologist, since failure to recognize these issues in children can result in poor outcomes.^2^,^3^ Both morbidity and mortality may be impacted by pulmonary problems following liver transplantation, which frequently necessitate critical care in the early postoperative phase. Pneumonia, pleural effusion, pulmonary edema, and atelectasis are the primary pulmonary consequences.^4^,^5^

One typical pulmonary sign of end-stage liver disease is pleural effusion. Hepatic artery and portal vein thrombosis, systemic infection, and allograft rejection are among the problems that cause the majority of post-OLT deaths, which happen within three months of OLT. Numerous studies indicate that between 40% to 80% of patients experience postoperative pulmonary problems.^8^ In our study, the incidence of pulmonary complications was 60%.

Frequent blood transfusions during LT, increased susceptibility to infection from immunosuppressants, and preoperative pulmonary pathology from liver cirrhosis (hepatothorax, hepatopulmonary syndrome, and portopulmonary syndrome) can all contribute to pulmonary problems following LT. Following OLT, pleural effusions can present with a wide range of clinical symptoms. Usually transudative in composition, these effusions are asymptomatic and temporary, and they are frequently treatable medically. But certain effusions don’t go away and need to be removed invasively.^9^,^10^

The pathogenic condition, which is typically localized in the right pleural cavity, results from peritoneal fluid leaking through the diaphragm. Liver transplantation is made more difficult by a number of pulmonary conditions that are seen in terminal liver illnesses and impact the pleura, lung parenchyma, and pulmonary arteries. This volume overload condition is still poorly understood, but it is believed to be primarily caused by diaphragmatic damage from prolonged retraction of the right hemidiaphragm during hepatectomy surgery, which causes fluid to move from the abdominal cavity into the pleural cavity, as well as increased hydrostatic pressure secondary to portal hypertension and disruption of the diaphragmatic lymphatic vessels.^11^-^16^ Pulmonary issues also arise as a result of the administration of immunosuppressives and the requirement for postoperative mechanical breathing. Subdiaphragmatic pathology, such as infection, hematoma, and biloma, as well as perioperative blood product infusion, hypoalbuminemia, and atelectasis, might also be the cause. Because of the possibility of surgical complications and hemodynamic instability, perioperative fluid management is still a difficult part of pediatric liver transplantation.^13^,^14^

Pleural effusion following liver transplantation is typically described as a self-limiting condition. Large amounts of pleural effusion, however, might affect respiratory function, particularly in young infants.^15^ Pleural effusion that develop in children after LT are minor, temporary, and most likely reactive. Almost all patients arrive in the intensive care unit with an endotracheal tube, although the extubation of stable patients is usually possible within 48 hours.

Increased respiratory demands from illness or airway blockage, pulmonary edema, pleural effusion, atelectasis, or elevated carbon dioxide generation can all result in the requirement for longer ventilatory support.^15^,^16^ Pleural effusion during paediatric LT was reasonably common (60%) according to our analysis, with the majority of these cases occurring in the right lung. The majority of pleural effusions heal on their own, although in this study, about 23.3% of patients needed therapeutic procedures such thoracentesis or ICD.

Furthermore, our study found that patients who developed pleural effusion requiring therapeutic intervention experienced prolonged intubations, extended oxygen dependency, longer intensive care unit (ICU) stays, and overall increased hospitalization time compared to those who did not require such interventions.^17^

The Pediatric End-Stage Liver Disease (PELD) score remains a reliable metric for assessing the severity of chronic liver disease and is predictive of both mortality and post-transplant survival in pediatric patients awaiting orthotopic liver transplantation (OLT). Patients with higher PELD scores often presented with broad collateral circulation and significant portal hypertension, in our experience, were often exacerbated by significant intraoperative hemorrhage, requiring larger volumes of fluid resuscitation during and after surgery. Although statistical significance was not reached, One finding is that patients with pleural effusion received more blood component transfusions than controls, increasing their theoretical risk of post-Transplant pleural effusion among candidates with high PELD scores. Moreover, poor nutritional status in such patients may promote higher rates of perioperative complications.^15^-^19^

Previous studies have shown a significant decrease in pulmonary complications following liver transplantation when intraoperative transfusions are maintained below 100 mL/kg/day and a negative fluid balance is achieved within the first three postoperative days.^2^ However, the use of high-dose methylprednisolone in the immediate post-transplant period to prevent acute Cellular rejection can also worsen fluid retention and make it especially hard to achieve a negative fluid balance during this critical period.^16^-^18^ In our cohort, about 23.3% of patients needed active intervention for pleural effusion. The main indications for intervention included clinical or radiologic evidence of respiratory compromise, and the need to rule out infectious pleural fluid. While pleural effusions ultimately resolved in all cases whether managed, either conservatively or with intervention-they were associated with increased oxygen requirements, longer stays in the ICU, and extended hospitalization.

Most of the children requiring OLT in our study were under five years of age and exhibited significant malnutrition. Hence, timely referral for transplantation and identification of pediatric cirrhotic patients is critically important for the minimization of postoperative complications, such as pleural effusion.

Although ten patients in our study succumbed postoperatively, none of the deaths were directly due to pleural effusion. Other contributing factors included graft dysfunction and infectious complications.

### Strength of this study:

The key strength of this study is its use of data from a single center, which allowed for the routine implementation of uniform clinical practices.

### Limitations:

Data was collected at one institution, the findings may reflect specific institutional practices, demographics, or protocols that differ from other centers and a moderate sample might lack the statistical power to detect smaller effect sizes or account for all possible confounding variables. However, we recognizing certain limitations, including underreporting and the fact that many patients were critically ill at the time of transplantation, which might have affected both intraoperative and postoperative outcomes. Consequently, further larger patient cohorts in prospective studies are needed to confirm our findings.

## CONCLUSION

Multiple risk factors are associated with the development of pleural effusion in pediatric liver transplant recipients. These common factors include malnutrition, low serum albumin, and total protein levels; decreased haemoglobin and fibrinogen levels; a high PELD score ≥ 18 at the time of liver transplantation; and excessive intraoperative fluid retention. These are believed to be significant predictors of pleural effusion after transplant. The implementation of preventive strategies, such as the optimization of nutritional and medical status pretransplant, may improve clinical outcome and possibly reduce mortality. Post liver transplant pleural effusion is associated with prolonged oxygen dependency, longer ICU stays, and extended hospitalization, particularly in patients requiring interventions. Early referral of paediatric cirrhotic patients to liver transplant centers is very important in order to reduce complications, including pleural effusion, and to reduce recovery time.
